# Unwrapping the problems: endoscopic repair of fundoplication fistula

**DOI:** 10.1055/a-2647-4476

**Published:** 2025-07-29

**Authors:** Michael Ma, Arvind J. Trindade, Petros C. Benias

**Affiliations:** 1 Division of Gastroenterology and Hepatology, Department of Medicine, Rutgers Health, Robert Wood Johnson Medical School, New Brunswick, United States

Fundoplication is the recommended surgical management for gastroesophageal reflux disease 1 . However, common postoperative complications such as dysphagia in up to 46% 2 , and rare complications such as esophagogastric fistula (EGF), can develop. Surgical revision is needed in approximately 11% of those undergoing fundoplication. We present a case of endoscopic management of EGF in the setting of prior fundoplication.

A 38-year-old patient with a history of Nissen fundoplication with subsequent takedown with myotomy and partial fundoplication experienced multiple food impactions requiring 38 endoscopies in the past 4 years. Prior endoscopy was notable for an EGF at the gastroesophageal junction, with the distal orifice in the area of the fundus. This fistula was leading to repeated food impactions that required endoscopic removal. Given the frequency of endoscopic interventions, the patient agreed to pursue endoscopic dissection of the plicated tissue forming the EGF and gastric peroral endoscopic myotomy (G-POEM) to manage gastroparesis.

The shelf of the EGF formed from the plicated tissue was dissected with a hybrid knife ( Video 1 ). The fistula was completely obliterated after complete dissection. G-POEM was also performed successfully and uneventful. An overall improvement in symptoms was noted following the procedure, and reflux disease was controlled on medication.

Esophagogastric fistula managed with endoscopic dissection of the plicated tissue shelf.Video 1

EGF is a rare complication of fundoplication and can cause a myriad of symptoms depending on the size and location of the fistulous tract. There are case reports on EGF but literature is limited, with refractory disease often necessitating surgical intervention 3 . We present a patient with a large symptomatic EGF causing dysphagia and food impaction. Endoscopic repair of the EGF successfully treated symptoms. As the shelf forming the fistulous tract was created by fundoplication and consisted of gastric tissue, it was safe to dissect and obliterate the fistulous tract.

Endoscopy_UCTN_Code_TTT_1AT_2AC

## Notes

Endoscopy E-Videos https://eref.thieme.de/e-videos
*E-Videos* is an open access online section of the journal *Endoscopy* , reporting on interesting cases and new techniques in gastroenterological endoscopy. All papers include a high-quality video and are published with a Creative Commons CC-BY license. Endoscopy E-Videos qualify for HINARI discounts and waivers and eligibility is automatically checked during the submission process. We grant 100% waivers to articles whose corresponding authors are based in Group A countries and 50% waivers to those who are based in Group B countries as classified by Research4Life (see: https://www.research4life.org/access/eligibility/ ). This section has its own submission website at https://mc.manuscriptcentral.com/e-videos .
